# Pyrrole Alkaloids from the Edible Mushroom *Phlebopus portentosus* with Their Bioactive Activities

**DOI:** 10.3390/molecules23051198

**Published:** 2018-05-17

**Authors:** Zhaocui Sun, Meigeng Hu, Zhonghao Sun, Nailiang Zhu, Junshan Yang, Guoxu Ma, Xudong Xu

**Affiliations:** Key Laboratory of Bioactive Substances and Resource Utilization of Chinese Herbal Medicine, Ministry of Education, Beijing Key Laboratory of Innovative Drug Discovery of Traditional Chinese Medicine (Natural Medicine) and Translational Medicine, Key Laboratory of Efficacy Evaluation of Chinese Medicine against Glycolipid Metabolic Disorders, State Administration of Traditional Chinese Medicine, Institute of Medicinal Plant Development, Peking Union Medical College and Chinese Academy of Medical Sciences, Beijing 100193, China; flydancingsun@163.com (Z.S.); humeigeng@outlook.com (M.H.); sun_zhonghao@126.com (Z.S.); nlzhu@implad.ac.cn (N.Z.); jsyang@implad.ac.cn (J.Y.)

**Keywords:** *Phlebopus portentosus*, edible mushroom, pyrrole alkaloids, neuroprotection

## Abstract

Seven pyrrole alkaloids, three of which are novel (phlebopines A–C (**1**–**3**)), were isolated from the fruiting bodies of the edible mushroom *Phlebopus portentosus*. Their structures were determined on the basis of spectroscopic data. All the isolated compounds were tested for their neuroprotective properties and acetylcholine esterase (AChE) inhibition activities. Compound **7** displayed remarkable neuroprotective effects against hydrogen peroxide (H_2_O_2_)-induced neuronal-cell damage in human neuroblastoma SH-SY5Y cells.

## 1. Introduction

Edible mushrooms are very popular with the public due to their pleasant taste, low energy, fat content, and health properties [1,2]. Additionally, the extractions of some edible mushrooms display various human health benefits, such as neuroprotective activity, antifatigue ability, improved immunity, cough relief, reduced sputum, and antitumor properties [3,4,5,6]. In recent years, edible mushrooms have attracted much attention from chemists and biologists because their components contain novel structures and diverse bioactivities. Terreumols with a rare 10-membered ring system display remarkable cyctoxicities against five human cancer cell lines, and were isolated from the fruiting bodies of edible mushroom *Tricholoma terreum* [5]. Matsutakone is an acetylcholinesterase inhibitor with an unprecedented polycyclic ring system derived from the edible mushroom *Tricholoma matsutake* [6].

**Figure 1:**
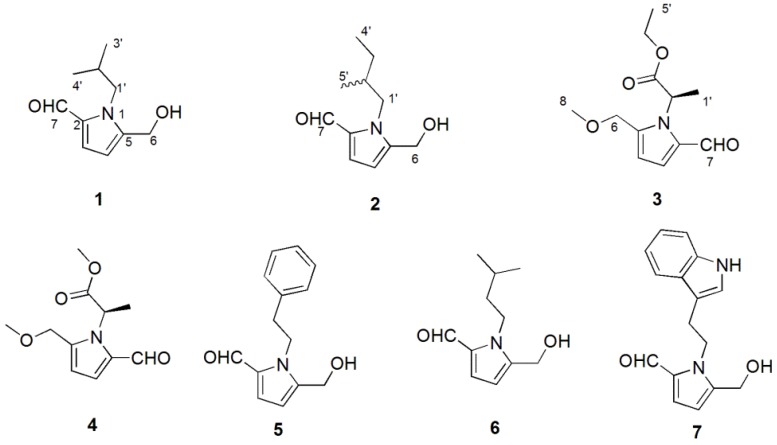
Structures of compounds **1**–**7**.

*Phlebopus portentosus* (Berk. and Broome) Boedijin is an edible mushroom with a wide distribution in tropical parts of China, especially in the Yunnan, Guangxi, and Hannan provinces [7]. It is very popular for its prized flavor and large black fruiting body, which is rich in protein, crude fat, polysaccharide, crude fiber, mineral elements, and amino acids [8,9]. However, the pharmacological activity and active material basis of this species have not been researched. In our screening program for the neuroprotective activity of edible mushrooms, the ethyl acetate extraction of *P. portentosus* exhibited moderate activity against H_2_O_2_ induced damage in SH-SY5Y neuroblastma cells. With the purpose of finding new neuroprotective compounds, we examined the AcOEt extraction of *P. portentosus*, and found three new pyrrole alkaloids, and phlebopines A–C (**1**–**3**), along with four known ones: 2-[2-formyl-5-(methoxymethyl)-1*H*-pyrrole-1-yl]propanoate (**4**) [10], inotopyrrole (**5**) [11], 1-isopentyl-2-formyl-5-hydroxy-methylpyrrole (**6**) [12], and inotopyrrole B (**7**) [13] (Figure 1). This paper reports the isolation and structural elucidation of the isolated pyrrole alkaloids, as well as their neuroprotection and acetylcholine esterase (AChE) inhibition.

## 2. Results

### 2.1. Structure Elucidation

**Figure 2:**
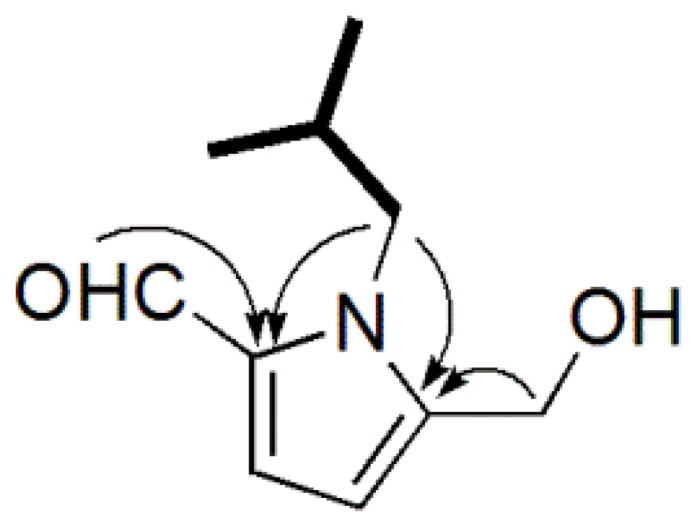
Key ^1^H-^1^H COSY (bolded items) and HMBC (arrows) correlations of compound **1**.

Compound **1**, was purified as a white powder, and has a molecular formula of C_10_H_15_NO_2_ which was deduced by a HRESIMS quasimolecular ion at *m*/*z* 204.0997 [M + Na]^+^ (calculated for C_10_H_15_NO_2_Na, 204.1000). It had an IR absorption band at 1647 cm^−1^, which suggested the presence of an unsaturated carbonyl group. An absorption band at 291 nm in the UV spectrum of compound **1** was indicative of a pyrrole-2-aldehyde moiety [14]. The ^1^H-NMR spectrum (Table 1, supplementary material Figure S1) displayed a set of two mutual coupled protons at *δ*_H_ 6.98 (1H, d, *J* = 4.2 Hz, H-3) and 6.23 (1H, d, *J* = 4.2 Hz, H-4), which indicated the presence of a typical 2,5-disubstituted pyrrole ring [15,16]. The presence of an aldehyde group in compound **1** was supported by the NMR signals at *δ*_H_ 9.44 (1H, s) and *δ*_C_ 179.5. Two oxygenated protons at *δ*_H_ 4.50 (s, 2H), together with the downfield methylene carbon signal at *δ*_C_ 55.6, suggested the existence of a hydroxymethyl group. ^1^H-NMR signals of *δ*_H_ 0.80 (6H, d, *J* = 7.2 Hz, overlapped, H-3′/4′), 2.00 (1H, m, H-2′), 4.11 (2H, d, *J* = 7.8 Hz, H-1′), and ^13^C-NMR APT (Table 1, supplementary material Figure S2) signals of *δ*_C_ 19.9 (overlapped), 30.1, and 51.9 indicated the presence of an isobutyl moiety, which was further confirmed by ^1^H-^1^H COSY correlations of H-3′/H-2′/H-1′ (Figure 2). In the HMBC spectrum (Figure 2), the aldehyde proton had a long-range correlation with C-2 (*δ*_C_ 132.4), while hydroxymethyl protons showed enhancements with C-5 (*δ*_C_ 144.5), which suggests that the two groups were located at C-2 and C-5 of the pyrrole ring, respectively. The isobutyl side chain was attached to the N atom on the basis of HMBC correlations from H-1′ (*δ*_H_ 4.11) to C-2 and C-5, together with the downfield methine carbon of C-1′ (*δ*_C_ 51.9). Thus, the entire structure of compound **1** was elucidated as 1-isobutyl-2-formyl-5-hydroxymethylpyrrole, and given the trivial name phlebopine A.

Compound **2** was obtained as a white powder with the molecular formula of C_11_H_17_NO_2_ based on the positive ion HRESIMS peak at *m*/*z* 218.1132 [M + Na]^+^ (calculated for C_11_H_17_NO_2_Na, 218.1157). The ^1^H- and ^13^C-NMR spectroscopic data (Table 1) of compound **2** were quite similar to those of compound **1** except for the additional methylene (*δ*_H_ 1.12, 1.25; *δ*_C_ 26.7). Further comparison of the NMR data between compounds **1** and **2** revealed that one methyl group at C-2′ in **1** was replaced by an ethyl group in compound **2**. The proton signals at *δ*_H_ 0.84 (t, *J* = 7.2 Hz, H-4′), 1.12 (1H, m, H-3′a), and 1.25 (1H, m, H-3′b), together with their HMBC correlations from H-4′ and H-3′, confirmed the deduction above. However, the absence of proper model compounds to use as references made the assignment of the absolute configuration at C-2′ unreliable. Therefore, compound **2** was identified as 1-(2-methybutyl)-2-formyl-5-hydroxyl-methylpyrrole, and given the trivial name phlebopine B.

Compound **3**, was obtained as a white amorphous powder, with its molecular formula assigned as C_12_H_17_NO_4_ on the basis of its positive HRESIMS (*m*/*z* 262.1034 [M + Na]^+^), implying 5 degrees of unsaturation. The ^1^H-NMR spectrum (Table 1) displayed 2-aldehyde-5-hdyroxymethyl-pyrrole unit signals at *δ*_H_ 6.95 (1H, d, *J* = 4.2 Hz, H-3) and 6.26 (1H, d, *J* = 4.2 Hz, H-4), 4.48 (1H, d, *J* = 13.2 Hz, Ha), 4.42 (1H, d, *J* = 13.2 Hz, Hb), and 9.43 (s), which indicated that compound **3** was an analogue of compound **1**. Other signals of methoxyl appeared at *δ*_H_ 3.31, ethoxyl at *δ*_H_ 1.21 (3H), 4.17 (2H), methyl at *δ*_H_ 1.70, and methine at *δ*_H_ 4.00. Except for the 2,5-disubstituted pyrrole moiety and one ester carbonyl (*δ*_C_ 170.1), the ^13^C-NMR APT (Table 1) showed another five carbon signals corresponding to the groups above. In the HMBC spectrum, the methyl signals at *δ*_H_ 1.70 (3H, s, H-1′) had correlations with C-2′ (*δ*_C_ 54.7) and C-3′ (*δ*_C_ 170.1), indicating the presence of a -CH(CH_3_)CO- unit. The downfield chemical shift of C-2′ (*δ*_C_ 54.7) as well as the HMBC correlations between H-2′ and C-2 suggested the -CH(CH_3_)CO- unit was attached to the N atom of pyrrole ring. Furthermore, the HMBC correlations between *δ*_H_ 3.31 (-OCH_3_) and C-6 (*δ*_C_ 65.8), *δ*_H_ 4.17 (-OCH_2_-) and C-3′ (*δ*_C_ 170.1) indicated the methoxyl and ethoxyl groups were located at C-6 and C-3′, respectively. The CD spectrum of compound **3** exhibited a negative Cotton effect at 330 (Δ ε 8.7) nm due to the conjugated pyrrole ring chromophore, suggesting an *R* absolute configuration for C-2′ [10]. As a result, compound **3** was established as 1-ethylpropionate-2-formyl-5-methoxylmethylpyrrole, and given the trivial name phlebopine C.

### 2.2. Bioactive Activity

All the compounds were evaluated for their neuroprotection and acetylcholine esterase (AChE) inhibition activities. The results (Table 2) showed that compound **7** could significantly attenuate SH-SY5Y cell damage induced by H_2_O_2_ with a 26.5% increase in cell survival over the H_2_O_2_ group at 10 μM, compared with the positive control *N*-acetyl-l-cysteine causing a 24.3% increase in cell viability over the H_2_O_2_ group at a concentration of 10 μM. Other compounds displayed moderate or mild activities, ranging from a 5.84% to 15.7% increase in cell survival over the H_2_O_2_ group at 10 μM. However, none of the compounds have significant inhibitory activity against AChE at the concentration of 10 μM.

## 3. Discussion

Seven alkaloids, containing the characteristics of a pyrrole ring and an aldehyde group, were isolated from the edible mushroom *Phlebopus portentosus*. Until now, nearly 200 naturally occurring alkaloids with a pyrrole-2-aldehyde moiety have been reported. These kind of alkaloids are widely distributed in plants, microorganisms, and marine invertebrates [16,17,18,19]. However, compounds **1**–**7** with the pyrrole-2-aldehyde group were first isolated from the edible mushroom *Phlebopus portentosus*. Meanwhile, three new pyrrole alkaloids have also enriched the chemical diversity of this kind of alkaloids. In addition, we investigated all the compounds for their neuroprotective properties and acetylcholine esterase (AChE) inhibition activities. In cell survival over the H_2_O_2_ group at 10 μM, compound **7** significantly attenuated SH-SY5Y cell damage induced by H_2_O_2_, noting a 26.5% increase, while other compounds displayed moderate or mild activities ranging from a 5.84% to 15.7% increase. Compounds **5** and **7** better attenuated SH-SY5Y cell damage induced by H_2_O_2_ than other compounds, which suggested that pyrrole alkaloids with another aromatic ring in their indole ring may affect the pharmacological activity regarding SH-SY5Y cell damage induced by H_2_O_2_. 

## 4. Materials and Methods

### 4.1. General Experimental Procedures

Optical rotation data were measured with a Perkin-Elmer 341 digital polarimeter (PerkinElmer, Norwalk, CT, USA). UV and IR data spectra were recorded on Shimadzu UV2550 and FTIR-8400S spectrometers (Shimadzu, Kyoto, Japan). CD spectra were obtained using a JASCO J-815 spectropolarimeter. NMR spectra were obtained using a Bruker AV III 600 NMR spectrometer with chemical shift values presented as δ values having TMS as the internal standard. HRESIMS was performed using an LTQ-Orbitrap XL spectrometer (Thermo Fisher Scientific, Boston, MA, USA). Column chromatography (CC) was performed using a silica gel (100–200 and 300–400 mesh, Qingdao Marine Chemical Plant, Qingdao, China). Precoated silica gel GF_254_ plates (Zhi Fu Huang Wu Pilot Plant of Silica Gel Development, Yantai, China) were used for TLC. All solvents used were of analytical grade (Beijing Chemical Plant, Beijing, China).

### 4.2. Fungal Material

The mushrooms of *P. portentosus* were collected from Haikou City, Hainan Province, China, in April 2017. The botanical identification of the mushroom was done by Professor Xi-long Zheng, Hainan Branch Institute of Medicinal Plant Development, Chinese Academy of Medical Sciences & Peking Union Medical College, where a voucher specimen (No. M20170425) was deposited.

### 4.3. Isolation and Purification of Compounds ***1**–**7***

The dried fruiting bodies of *P. portentosus* (0.5 kg) were extracted with EtOAc twice. The solvents were filtrated and evaporated in vacuo to give the total extract (43 g), and this residue was subjected to column chromatography (CC) over silica gel (100–200 mesh) eluted with a gradient of CH_2_Cl_2_-MeOH (0:1→1:0) to obtain six fractions (A–F). Fraction C (8.2 g) was subjected to chromatography repeatedly over silica gel CC eluting with CH_2_Cl_2_-MeOH (80:0, 60:1, 40:1, 20:1, 10:1, *v*/*v*), and was finally purified by semipreparative HPLC (MeOH-H_2_O/80:20) to give compounds **1** (3.4 mg), **2** (2.7 mg), and **6** (8.0 mg). Fraction D (1.3 g) was subjected to chromatography using ODS MPLC elution with MeOH-H_2_O (30:70; 90:10; 100:0, *v*/*v*), to yield three fractions (Fr. D1-3), and Fr. D2 was separated through semipreparative HPLC using a mobile phase of MeOH-H_2_O (85:15, *v*/*v*) to afford compounds **3** (2.1 mg) and **4** (1.8 mg). Similarly, fraction E (2.9 g) was isolated through ODS MPLC elution with MeOH-H_2_O (30:70; 90:10; 100:0, *v*/*v*), and prepared by semipreparative HPLC to give compounds **5** (12.4 mg) and **7** (9.1 mg). 

### 4.4. Characterization of Compounds ***1**–**3***

phlebopine A (**1**), White powder (MeOH); UV (MeOH) λ_max_ (logε) 291 (3.52) nm; IR (film) ν_max_ 3430, 2928, 2854, 2735, 1647 cm^–1^; ^1^H- and ^13^C-NMR data (DMSO-*d_6_*), see (Table 1); HRESIMS *m*/*z* 204.0997 [M + Na]^+^. (Calculated for. 204.1000, C_10_H_15_NO_2_Na).

phlebopine B (**2**), White powder (MeOH); UV (MeOH) λ_max_ (logε) 294 (3.76) nm; IR (film) ν_max_ 3428, 2935, 2841, 2732, 1650 cm^–1^; ^1^H- and ^13^C-NMR data (DMSO-*d*_6_), see (Table 1); HRESIMS *m*/*z* 218.1132 [M + Na]^+^. (Calculated for. 218.1157, C_11_H_17_NO_2_Na).

phlebopine C (**3**), White powder (MeOH); UV (MeOH) λ_max_ (logε) 292 (3.93) nm; IR (film) ν_max_ 3368, 2954, 2830, 2728, 1672 cm^–1^; CD (MeOH): 330 (Δ ε −8.7); ^1^H- and ^13^C-NMR data (CDCl_3_), see (Table 1); HRESIMS *m*/*z* 262.1034 [M + Na]^+^. (Calculated for. 262.1055, C_12_H_17_NO_4_Na).

### 4.5. Neuroprotective Activity Assay

The neuroprotective activity was tested against H_2_O_2_-induced injury in SH-SY5Y neuroblastoma cells according to the reported protocol [20,21]. Cells were maintained at 37 °C in a humidified atmosphere containing 5% CO_2_. In 96-well plates, cells were seeded at a density of 2 × 10^5^ cells/mL in MEM/F12 medium supplemented with 10% (*v*/*v*) fetal bovine serum. Then, cells were incubated with the serum-free MEM/F12 medium substituting the original medium after 24 h. Test compounds and the positive control *N*-acetyl-l-cysteine were dissolved in DMSO to prepare 10^−2^ M stock solutions and then diluted to the corresponding concentrations with the cell culture medium. Cells were incubated with test compounds for 2 h prior to treatment with 10 μM. H_2_O_2_ for another 24 h without changing the culture medium. 10 μL of MTT (2 mg/mL, Sigma, Tokyo, Japan, purity: 98%) was then added to each well and incubated at 37 °C for 3 h. The cells were finally lysed with 100 μL of DMSO, and the amount of MTT formazan was measured at 490 nm using a microplate reader (M200, TECAN, Austria GmbH, Vienna, Austria).

### 4.6. Acetylcholinesterase Inhibitory Assay

Inhibition of acetylcholinesterase activity was measured by Atanasova’s spectrophotometric method with slight modifications [22,23]. The hydrolysate of acetylthiocholine, which reacts with 5,5′-dithiobis-(2-nitrobenzoic acid) (DTNB), can be detected at 405 nm. On the 96-well plate was placed: 50 μL of AChE in buffer phosphate (PH 7.6) and 50 μL of the sample dissolved in the same buffer were added to the wells. The plates were incubated for 30 min at room temperature before the addition of the substrate solution (0.5 M DTNB, 0.6 mM ATCI in buffer, pH 7.6). The absorbances were read at 405 nm using a microplate reader. 

## Figures and Tables

**Table 1:** NMR spectral data of **1**–**3** (600 MHz for ^1^H-NMR and 150 MHz for ^13^C-NMR).

No.	1 ^a^		2 ^a^		3 ^b^	
	*δ*_C_, Type	*δ*_H_ (*J* in Hz)	*δ*_C_, Type	*δ*_H_ (*J* in Hz)	*δ*_C_, Type	*δ*_H_ (*J* in Hz)
1	--	--	--	--	--	--
2	132.4, C	--	132.5, C	--	132.5, C	--
3	124.6 ^c^, CH	6.98, d (4.2)	124.7 ^c^, CH	6.98, d (4.2)	125.0, CH	6.95, d (4.2)
4	110.0, CH	6.23, d (4.2)	110.0, CH	6.22, d (4.2)	111.8, CH	6.26, d (4.2)
5	144.5, C	--	144.6, C	--	139.2 ^c^, C	--
6	55.6, CH_2_	4.50, s	55.6, CH_2_	4.49, s	65.8, CH_2_	4.48, d (13.2); 4.42, d (13.2)
7	179.5, CH	9.44, s	179.5, CH	9.44, s	178.9, CH	9.43, s
8	--	--	--	--	57.6, CH_3_	3.31, s
1′	51.9, CH	4.11, d (7.8)	50.8, CH_2_	4.12, m; 4.20, m	17.7, CH_3_	1.70, d (7.2)
2′	30.1, CH	2.00, m	36.5, CH	1.79, m	54.7, CH	4.00, m
3′	19.9, CH_3_	0.80, d (7.2)	26.7, CH_2_	1.12, m; 1.25, m	170.1 ^c^, C	--
4′	19.9, CH_3_	0.80, d (7.2)	11.7, CH_3_	0.84, t (7.2)	61.5, CH_2_	4.17, m
5′			16.7, CH_3_	0.73, d (6.6)	14.1, CH_3_	1.21, t (7.2)

^a^ Spectra data were recorded in DMSO-*d*_6_; ^b^ Spectra data were recorded in CDCl_3_; ^c^ Data were observed from HSQC and HMBC spectra.

**Table 2:** Effect of isolated compounds on SH-SY5Y cell damage induced by H_2_O_2_.

Compounds	Cell Viability (% of Control)
**1**	54.57 ± 0.97 ^a^
**2**	57.62 ± 3.42
**3**	60.27 ± 2.38
**4**	62.58 ± 3.32
**5**	64.43 ± 1.57
**6**	59.31 ± 2.01
**7**	75.23 ± 1.76
*N*-acetyl-l-cysteine ^b^	73.03 ± 1.49
H_2_O_2_ control	48.73 ± 1.08
Blank control	100.00 ± 2.43

^a^ Values are means ± SD of triplicate experiments; ^b^ Positive control substance.

## Supplementary Materials

Supplementary data associated with this article can be found, in the online version.

## References

[1] Longvah T., Deosthale Y.G. (1998). Compositional and nutritional studies on edible wild mushroom from northeast India. Food Chem..

[2] Manzi P., Aguzzi A., Pizzoferrato L. (2001). Nutritional value of mushrooms widely consumed in Italy. Food Chem..

[3] Wani B.A., Bodha R.H., Wani A.H. (2010). Nutritional and medicinal importance of mushrooms. J. Med. Plant Res..

[4] Kawagishi H., Shimada A., Shirai R., Okamoto K., Ojima F., Sakamoto H., Ishiguro Y., Furukawa S. (1994). Erinacines, A, B and C, strong stimulators of nerve growth factor (NGF)-synthesis, from the mycelia of Hericium erinaceum. Tetrahedron Lett..

[5] Yin X., Feng T., Li Z.H., Dong Z.J., Li Y., Liu J.K. (2013). Highly oxygenated meroterpenoids from fruiting bodies of the mushroom Tricholoma terreum. J. Nat. Prod..

[6] Zhao Z.Z., Chen H.P., Wu B., Zhang L., Li Z.H., Feng T., Liu J.K. (2017). Matsutakone and Matsutoic Acid, two (nor)steroids with unusual skeletons from the edible mushroom Tricholoma matsutake. J. Organ. Chem..

[7] Ji K.P., Cao Y., Zhang C.X., He M.X., Liu J., Wang W.B., Wang Y. (2011). Cultivation of *Phlebopus portentosus* in southern China. Mycol. Prog..

[8] Ji K., Zhang C., Zeng Y., Liu C., He M., Wang W. (2007). Artificial fungal colony and its fruiting of *Phlebopus portentosus* (Boletaceae) in pot. Acta Botanica Yunnanica.

[9] Yang Z., Zang M. (2003). Tropical Affinities of Higher Fungi in Southern China. Acta Botanica Yunnanica.

[10] Joung Youn U., Kil Y.S., Nam J.W., Jin Lee Y., Kim J., Lee D., Lee J.H., Seo E.K. (2013). New Pyrrole Alkaloids with Bulky N-Alkyl Side Chains Containing Stereogenic Centers from Lycium chinense. Helv. Chim. Acta.

[11] Zhan Z.J., Shan W.G., Bai H.B., Zhang L.Y. (2014). A new alkaloid from the mycelium of *Inonotus obliquus*. J. Chem. Res..

[12] Yang J.J., Yu D.Q. (1990). Synthesis of ganoderma alkaloid A and B. Yao Xue Xue Bao.

[13] Shan W.-G., Wang Y., Ma L.-F., Zhan Z.-J. (2017). A new pyrrole alkaloid from the mycelium of *Inonotus obliquus*. J. Chem. Res..

[14] Li M., Xiong J., Huang Y., Wang L.-J., Tang Y., Yang G.-X., Liu X.-H., Wei B.-G., Fan H., Zhao Y. (2015). Xylapyrrosides A and B, two rare sugar-morpholine spiroketal pyrrole-derived alkaloids from *Xylaria nigripes*: Isolation, complete structure elucidation, and total syntheses. Tetrahedron.

[15] Shigematsu H., Kurata T., Kato H., Fujimaki M. (1971). Formation of 2-(5-Hyclroxymethyl-2-formylpyrrol-1-yl) alkyl Acid Lactones on Roasting Alkyl-α-amino Acid with d-Glucose. Agric. Biol. Chem..

[16] Xiong J., Huang Y., Wu X.Y., Liu X.H., Fan H., Wang W., Zhao Y., Yang G.X., Zhang H.Y., Hu J.F. (2016). Chemical constituents from the fermented mycelia of the medicinal fungus *Xylaria nigripes*. Helv. Chim. Acta.

[17] Xiong L., Peng C., Xie X.F., Guo L., He C.J., Geng Z., Wan F., Dai O., Zhou Q.M. (2012). Alkaloids isolated from the lateral root of *Aconitum carmichaelii*. Molecules.

[18] Compagnone R.S., Oliveri M.C., Piãna I.C., Marques S., Rangel H.R., Dagger F., Suárez A.I., Gómez M. (1999). 5-alkylpyrrole-2-carboxaldehydes from the caribbean sponges mycale microsigmatosa and desmapsamma anchorata. Nat. Prod. Lett..

[19] Mao S.C., Liu Y., Morgan J.B., Jekabsons M.B., Zhou Y.D., Nagle D.G. (2009). Lipophilic 2,5-disubstituted pyrroles from the marine sponge mycale sp. inhibit mitochondrial respiration and hif-1 activation. J. Nat. Prod..

[20] Tang Y., Fu Y., Xiong J., Li M., Ma G.-L., Yang G.-X., Wei B.-G., Zhao Y., Zhang H.-Y., Hu J.-F. (2013). lycodine-type alkaloids from Lycopodiastrum casuarinoides. J. Nat. Prod..

[21] Taveira M., Sousa C., Valentão P., Ferreres F., Teixeira J.P., Andrade P.B. (2014). Neuroprotective effect of steroidal alkaloids on glutamate-induced toxicity by preserving mitochondrial membrane potential and reducing oxidative stress. J. Steroid Biochem. Mol. Biol..

[22] Atanasova M., Stavrakov G., Philipova I., Zheleva D., Yordanov N., Doytchinova I. (2015). Galantamine derivatives with indole moiety: Docking, design, synthesis and acetylcholinesterase inhibitory activity. Bioorganic Med. Chem..

[23] Mukherjee P.K., Kumar V., Mal M., Houghton P.J. (2007). Acetylcholinesterase inhibitors from plants. Phytomedicine.

