# Supplementary material for: Pyrrole Alkaloids from the Edible Mushroom Phlebopus portentosus with Their Bioactive Activities
Source: Molecules. 2018 May 17;23(5):1198. doi: 10.3390/molecules23051198 (PMC6100406; doi:10.3390/molecules23051198)
Supplement: Supplementary file 1 [file molecules-23-01198-s001.pdf]

Pyrrole Alkaloids from the Edible Mushroom *Phlebopus portentosus* with Their Bioactive Activities

Zhaocui Sun †, Meigeng Hu †, Zhonghao Sun, Nailiang Zhu, Junshan Yang, Guoxu Ma * and Xudong Xu *

Key Laboratory of Bioactive Substances and Resource Utilization of Chinese Herbal Medicine, Ministry of Education, Beijing Key Laboratory of Innovative Drug Discovery of Traditional Chinese Medicine (Natural Medicine) and Translational Medicine, Key Laboratory of Efficacy Evaluation of Chinese Medicine against Glycolipid Metabolic Disorders, State Administration of Traditional Chinese Medicine,
Institute of Medicinal Plant Development, Peking Union Medical College and Chinese Academy of Medical Sciences, Beijing 100193, China; flydancingsun@163.com (Z.S.); humeigeng@outlook.com (M.H.);
sun_zhonghao@126.com (Z.S.); nlzhu@implad.ac.cn (N.Z.); jsyang@implad.ac.cn (J.Y.)

* Correspondence: mgxfl8785@163.com (G.M.); xdxu2012@163.com (X.X.); Tel.: +86-010-5783-3296 (G.M.)

† These authors contributed equally to this work.

Abstract: Seven pyrrole alkaloids, three of which are novel (phlebopines A–C (**1**–**3**)), were isolated from the fruiting bodies of the edible mushroom *Phlebopus portentosus*. Their structures were determined on the basis of spectroscopic data. All the isolated compounds were tested for their neuroprotective properties and acetylcholine esterase (AChE) inhibition activities. Compound **7** displayed remarkable neuroprotective effects against hydrogen peroxide (H_2O_2)-induced neuronal-cell damage in human neuroblastoma SH-SY5Y cells.

Keywords: *Phlebopus portentosus*; edible mushroom; pyrrole alkaloids; neuroprotection

---

List of Figures S1-S13

Figure S1. ^{1}H-NMR (600 MHz, DMSO-d_6) spectrum of the new compound **1**

Figure S2. ^{13}C-APT (150 MHz, DMSO-d_6) spectrum of the new compound **1**

Figure S3. HSQC spectrum of the new compound **1**

Figure S4. HMBC spectrum of the new compound **1**

Figure S5. ^{1}H-^{1}H COSY spectrum of the new compound **1**

Figure S6. ^{1}H-NMR (600 MHz, DMSO-d_6) spectrum of the new compound **2**

Figure S7. ^{13}C-APT (150 MHz, DMSO-d_6) spectrum of the new compound **2**

Figure S8. HSQC spectrum of the new compound **2**

Figure S9. HMBC spectrum of the new compound **2**

Figure S10. ^{1}H-NMR (600 MHz, $CDCl_3$) spectrum of the new compound **3**

Figure S11. ^{13}C-APT (150 MHz, $CDCl_3$) spectrum of the new compound **3**

Figure S12. HSQC spectrum of the new compound **3**

Figure S13. HMBC spectrum of the new compound **3**

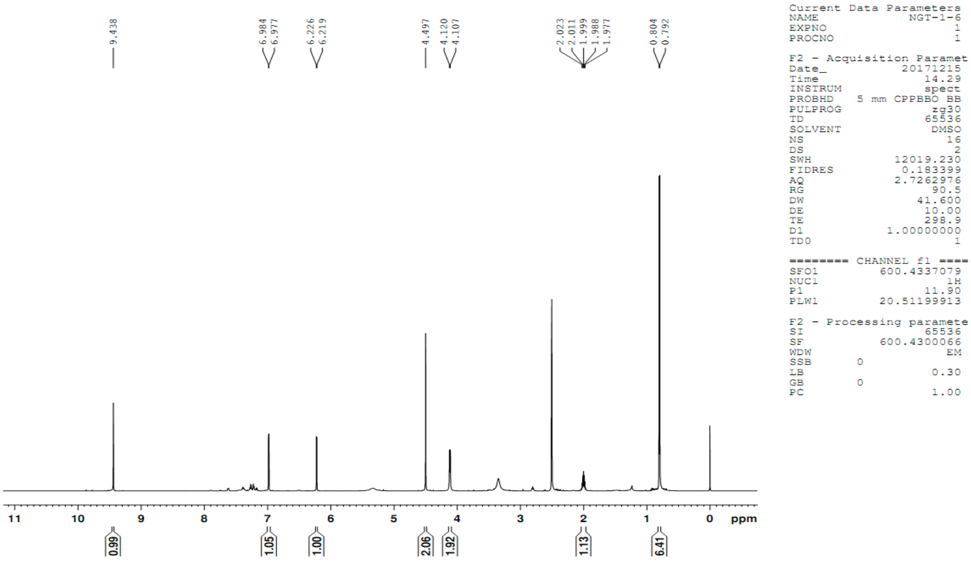

Figure S1. ¹H-NMR (600 MHz, DMSO-d_6) spectrum of the new compound **1.**

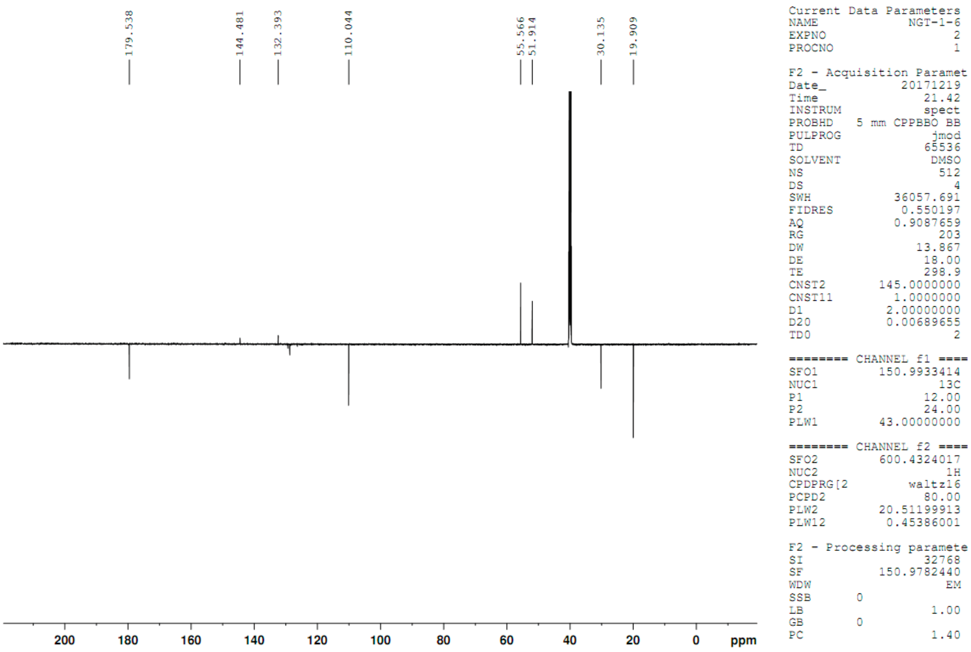

Figure S2. ¹³C-APT (150 MHz, DMSO-d_6) spectrum of the new compound **1.**

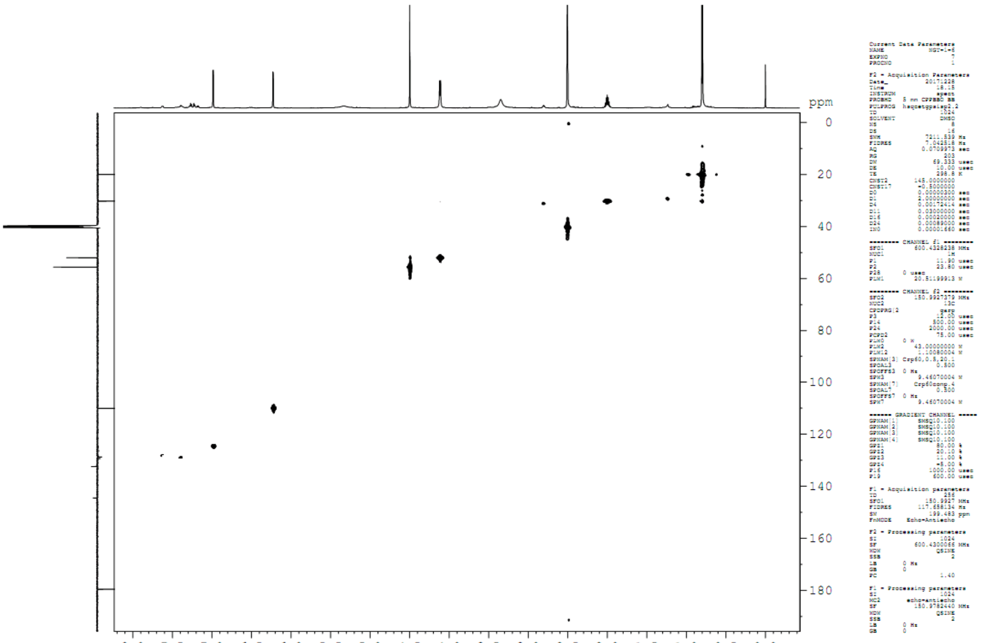

Figure S3. HSQC spectrum of the new compound **1.**

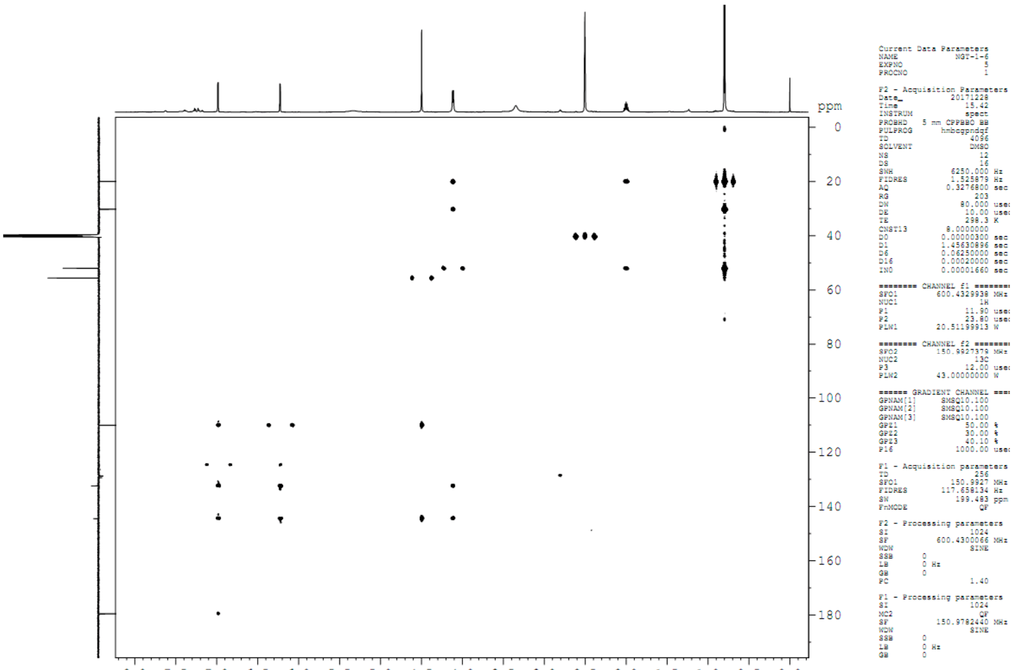

Figure S4. HMBC spectrum of the new compound **1.**

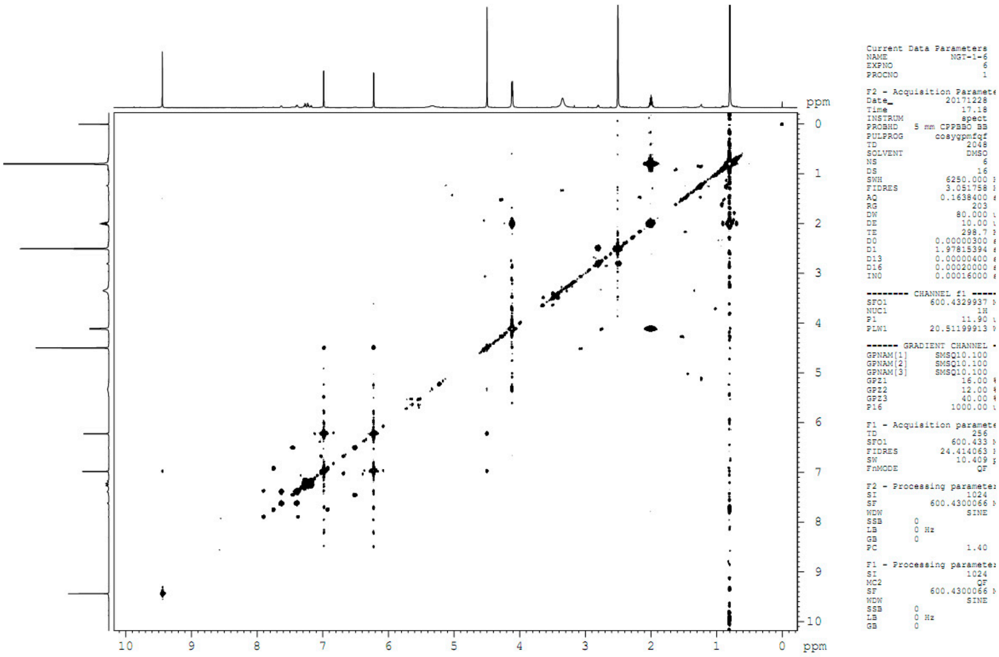

Figure S5. ^{1}H-^{1}H COSY spectrum of the new compound **1.**

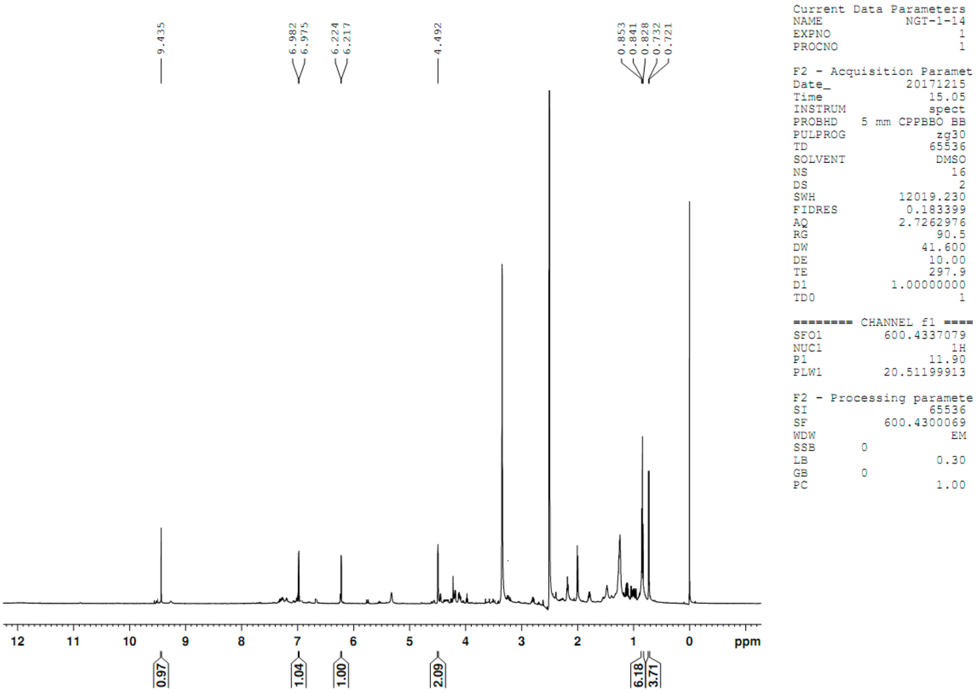

Figure S6. ^{1}H-NMR (600 MHz, DMSO-d_6) spectrum of the new compound **2.**

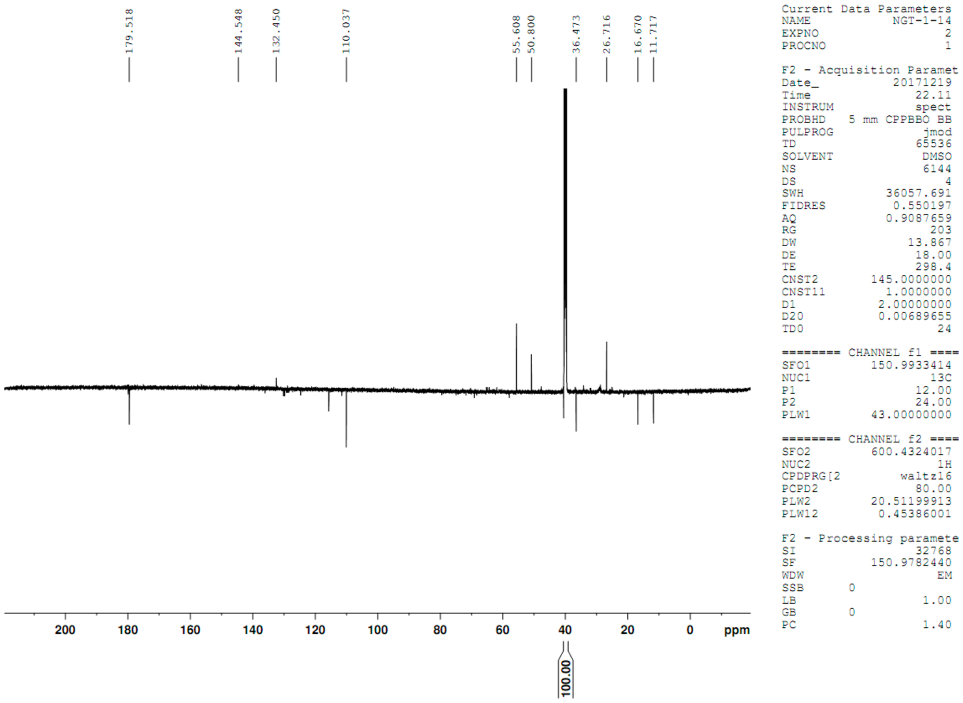

Figure S7. ^{13}C-APT (150 MHz, DMSO-d_6) spectrum of the new compound **2.**

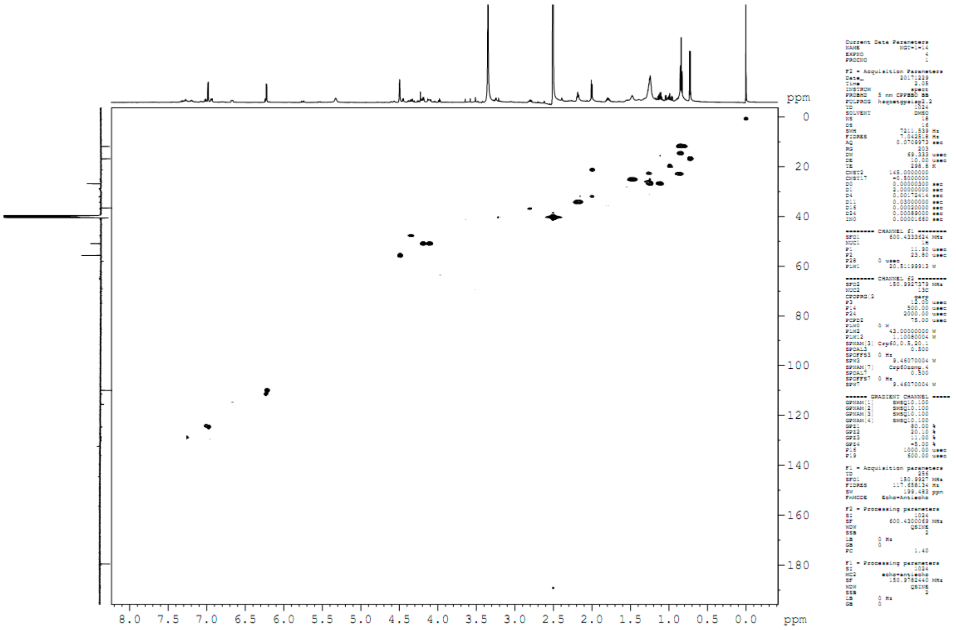

Figure S8. HSQC spectrum of the new compound **2.**

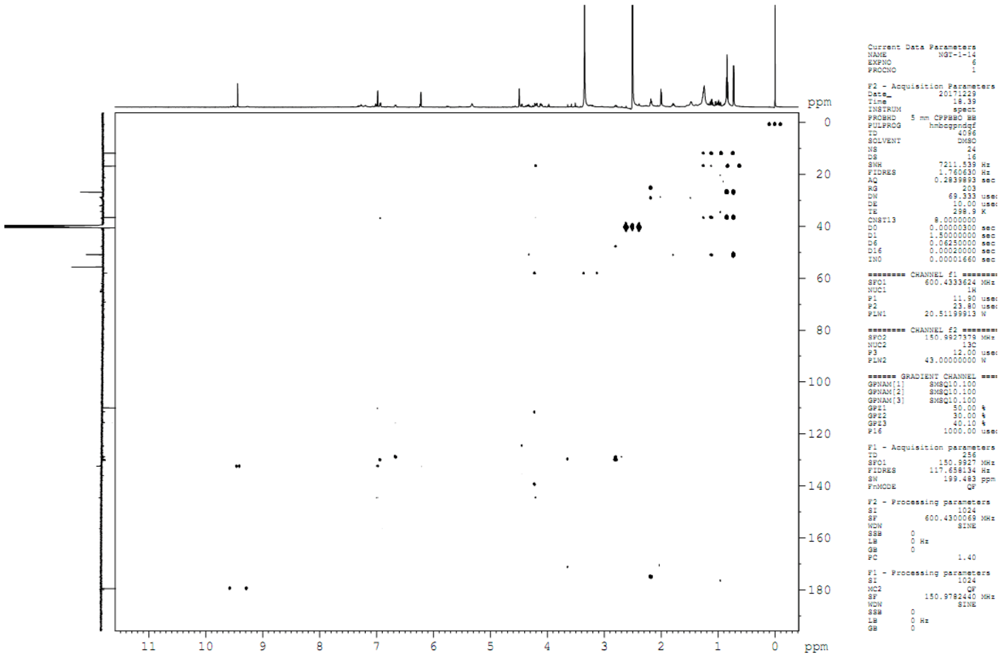

Figure S9. HMBC spectrum of the new compound **2.**

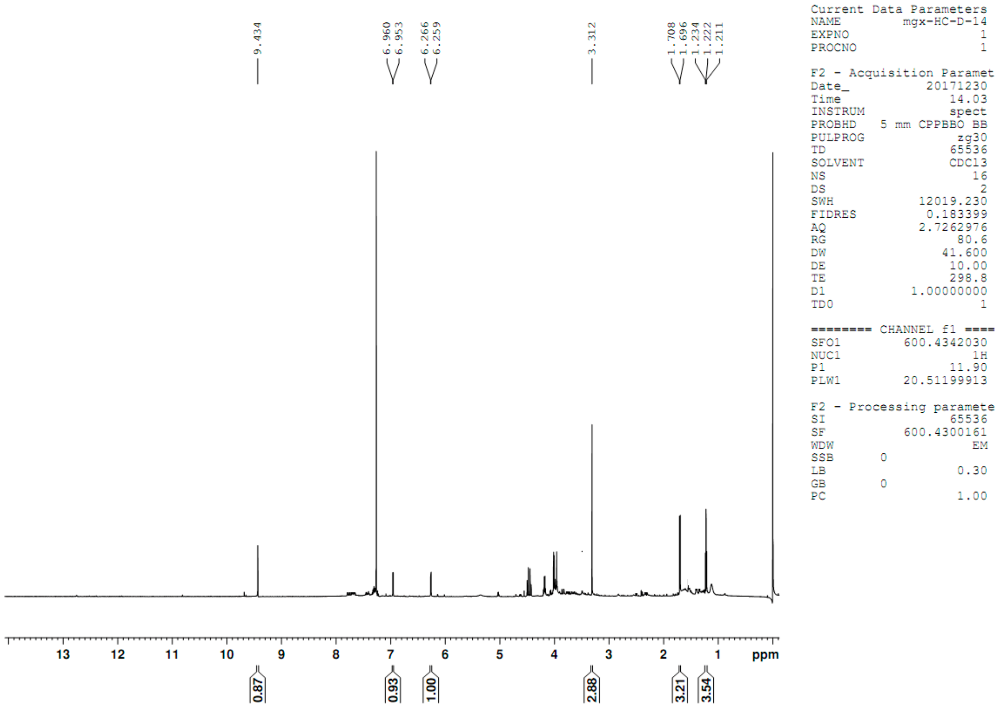

Figure S10. ^{1}H-NMR (600 MHz, $CDCl_3$) spectrum of the new compound **3.**

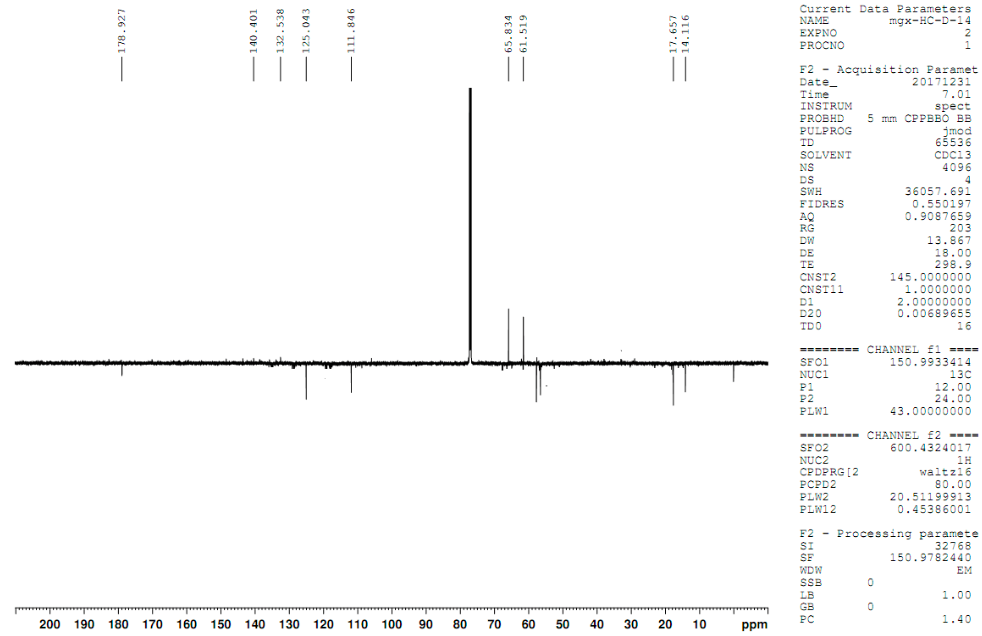

Figure S11. ^{13}C-APT (150 MHz, $CDCl_3$) spectrum of the new compound **3.**

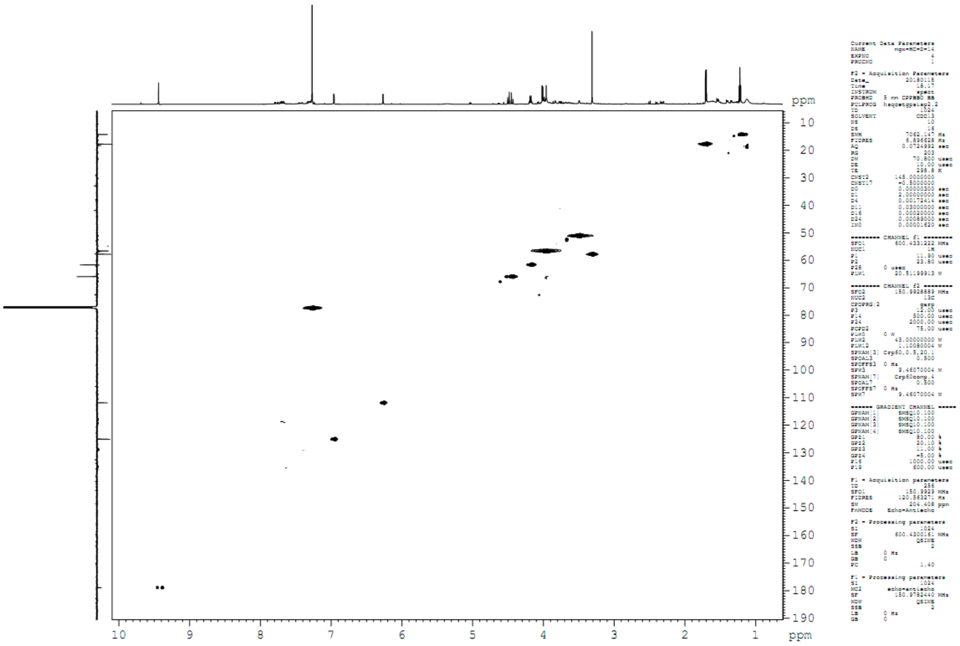

Figure S12. HSQC spectrum of the new compound **3.**

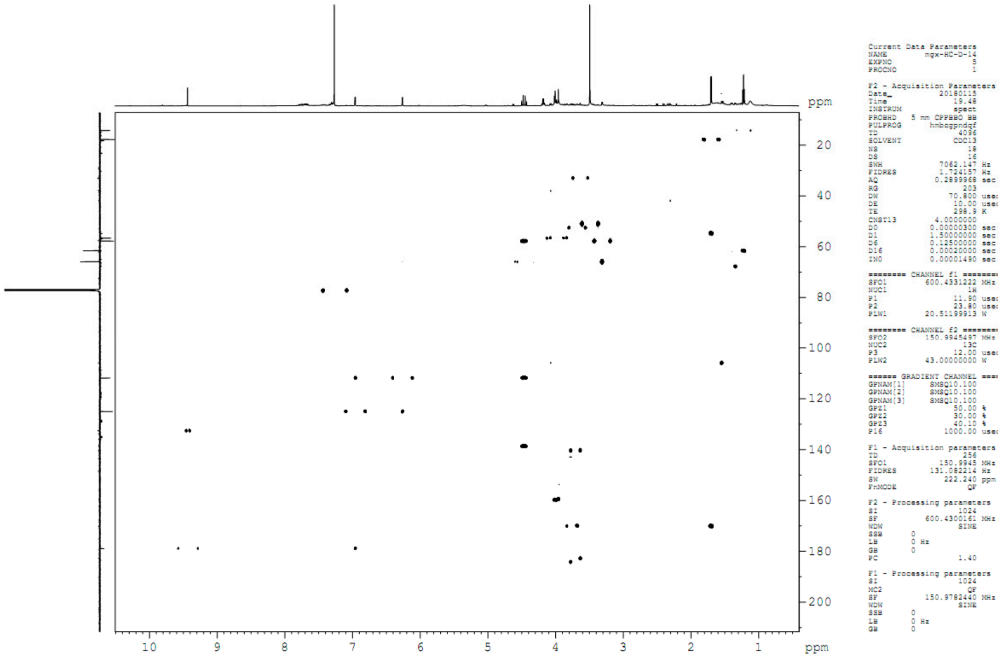

Figure S13. HMBC spectrum of the new compound **3.**
